# Pregnancy after renal transplantation: a retrospective study at the military hospital of Tunis from 1992 to 2011

**DOI:** 10.11604/pamj.2017.28.137.6287

**Published:** 2017-10-13

**Authors:** Ben Haj Hassine Amine, Siala Haythem, Harzallah Kais, Rachdi Radhouane

**Affiliations:** 1 Department of Gynecology and Obstetrics, Principal Military Hospital of Instruction of Tunis, Tunisia; 2 Organ Transplant Unit - Principal Military Hospital of Instruction of Tunis, Tunisia

**Keywords:** Renal graft, pregnancy, pre-eclampsia, intrauterine growth retardation

## Abstract

**Introduction:**

Our study objective was to analyze the optimum conditions for pregnancy in kidney transplanted women. For that, we conducted a retrospective study was from 1992 to April 2011 about 17 pregnancies in 12 kidney transplanted patients followed in the Department of Obstetrics and Gynecology and Organ Transplant Unit of the Military Hospital of Tunis.

**Methods:**

We studied nephrological parameters and obstetric pathologies encountered during pregnancy and the potential impact of pregnancy on graft. Our main outcome measures were: time between renal transplantation and conception, birth of a living child, renal graft defect.

**Results:**

The mean age at the time of renal transplantation was 30.11 years. The average age at the time of conception is 34.23 years. The average time between renal transplantation and the occurrence of pregnancy was 46.94 months. More than 40% of pregnancies were not planned. Of the 17 pregnancies, 12 have advanced beyond the first trimester with 91.6% resulting in the birth of a living child. Toxemia was found in 60% of cases, low birth weight in 50%, preterm in 30% and intrauterine growth retardation in 20% of cases. Cesarean section was indicated in all cases. Graft survival was 90% with a mean of 6 years after delivery.

**Conclusion:**

Pregnancy in kidney transplanted patients is a high-risk pregnancy, but pregnancy does not appear to affect graft function through certain conditions.

## Introduction

Kidney transplantation is undoubtedly a turning point in the lives of patients suffering from renal failure. In addition to improving the quality of life and prognosis, it is a legitimate hope for all those who want to conceive. Recovery of renal function is followed by the restoration of endocrine function allowing pregnancy. Motherhood has long been not recommended for kidney transplanted women by fear of deleterious effects on graft. Nowadays, under certain conditions, pregnancy in kidney graft is possible.

## Methods

We conducted a retrospective study was from 1992 to April 2011 about 17 pregnancies in 12 kidney transplanted patients followed in the Department of Obstetrics and Gynecology and Organ Transplant Unit of the Military Hospital of Tunis. We studied the terms of nephrological and obstetrical surveillance, pathologies appeared during pregnancy, the effects of pregnancy on graft and the impact of treatment received during pregnancy on the fetus.

## Results

**Figure 1:**
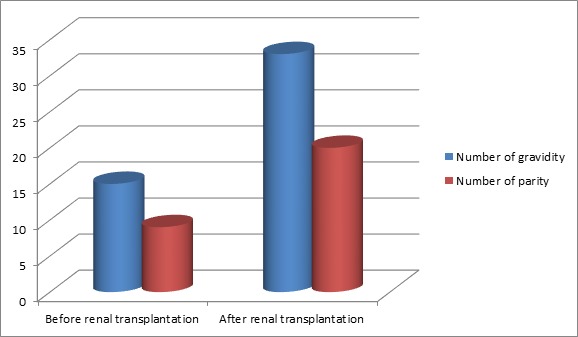
Improved fertility after renal transplantation

We collected data on 17 pregnancies in 12 kidney transplanted patients from 1992 to April 2011. During the same period, 52 women in age of procreation underwent kidney transplant at the Military Hospital of Tunis, a percentage of 23.07% of transplanted kidney who had at least one pregnancy. The kidney disease causing chronic hemodialysis setting was: tubulointerstitial nephropathy (5 cases), chronic glomerular nephropathy (3 cases), vascular nephropathy (3 cases) and indeterminate in one case. It is important to note that a previous pregnancy was the cause of the deterioration of renal function prior to renal transplantation in two patients. The mean duration of dialysis before transplantation was 68.83 months with extremes of 26 and 132 months. The mean age at transplantation was 30.11 years with extremes of 22 and 39 years. After kidney transplantation, the number of parity and gestity increased. The number of pregnancies has increased after renal transplantation (57.14%). Fertility has improved with improved renal function as evidenced by Figure 1.

All patients were informed of the need to wait a period of one year between renal transplantation and pregnancy. Termination of pregnancy was proposed in 4 cases for non-compliance of this period and was refused by all patients. Patients receiving mycophenolate mofetil have been informed of its teratogenic effects. Mycophenolate mofetil was stopped in 6 patients when the desire for pregnancy was formulated. The switch to azathioprine has been under effective contraception for 2 months. One patient was undergoing induction of ovulation (05 months prior to pregnancy). Renal function was stable before conception in all cases. The average time between renal transplantation and the occurrence of pregnancy was 46.94 months with extremes of 4 months and 18 days to 132 months. Different immunosuppressive protocols received before and during pregnancy are detailed in Table 1.

**Table 1:** The immunosuppressive therapy prior to and during pregnancy

Patient number	TT before pregnancy	TT after pregnancy
1	CsA+AZA+PDN	CsA+PDN^+^
2	CsA+PDN	CsA+PDN
3	CsA+AZA+PDN	CsA+AZA+PDN
4	CsA+AZA+PDN	CsA+AZA+PDN
5	CsA+AZA+PDN	CsA+AZA+PDN
6	CsA+AZA+PDN	CsA+AZA+PDN
7	MMF+PDN+FK506	AZA+PDN +FK506
8	MMF +PDN	AZA+PDN
9	MMF+CsA+PDN	CsA+AZA+PDN
10	MMF+CsA	CsA+AZA
11	MMF+PDN+ FK506	AZA+PDN+ FK506
12	MMF+PDN +FK506	AZA+PDN+ FK506

**TT**: Treatment, **Csa**: cyclosporine, **AZA**: azathioprine, **PDN**: prednisone

**MMF**: mycophenolate mofetil, **FK506** tacrolimus.

Stop AZA for cytolysis at 12 weeks of gestation

**Figure 2:**
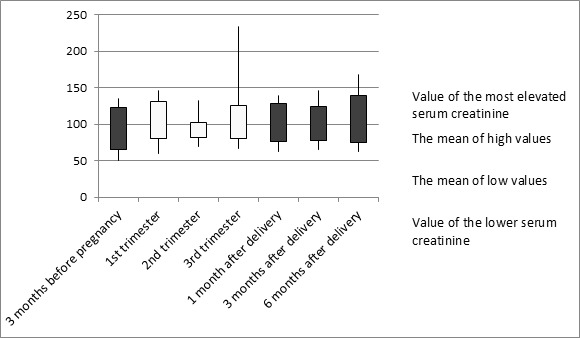
Evolution of serum creatinine before, during and after pregnancy

Of 17 pregnancies, 11 resulted in the birth of a living child, 3 were medically suspended and 3 were terminated spontaneously (2 miscarriages and one fetal death in utero). The live birth rate was 64.7%. There were no cases of ectopic pregnancy. In 2 cases, termination of pregnancy was indicated for unstable renal function at the time of the diagnosis of pregnancy. In pregnancies reaching at least the second trimester, we noted during follow-up a mean weight gain of 7100 grams, with a range of 5000 grams to 10000 grams. Six patients had chronic hypertension before pregnancy, treated with beta blockers. Two patients had pre-eclampsia. They developed proteinuria from the 3rd trimester (respectively 6th month and 7th month of pregnancy) without other signs of preeclampsia. Monitoring of renal function was performed in all patients. Serum creatinine was measured 3 months before pregnancy, during pregnancy and 1 month, 3 months and 6 months after delivery (Figure 2). During pregnancy, renal function remained stable in 75% of patients. Three patients had renal dysfunction, the cause was related to a toxemia in 2 cases. One patient developed renal dysfunction in late pregnancy which led to induce labor. The delivery was by caesarean section. The determination of plasma urea was done every month. The evolution was similar to that of serum creatinine. Before pregnancy, 24 hours proteinuria was zero in all cases. During pregnancy, two patients developed significant proteinuria respectively in the 24th and 29th week of gestation. Glycemia was measured every month. During pregnancy, fasting glycemia was stable in all cases. The provoked-glycemia by 50 grams of glucose was realized between the 24th and 32nd week of gestation to diagnose gestational diabetes. It was normal in all cases. None of our patients had diabetes before pregnancy. One patient developed type 2 diabetes six years after childbirth. Anemia during pregnancy occurred in 4 cases. Iron treatment has been strengthened. No patient received treatment with erythropoietin. One patient had an infection of the lowest urinary tract (E.Coli) in the first month of pregnancy treated with cefixime. The liver parameters were normal in all cases except for one patient. The occurrence of hepatic cytolysis in the 12^th^ week of gestation required discontinuation of azathioprine.

The dosage of immunosuppressant drugs was adapted according to their residual rate. The determination of residual rate of cyclosporine and tacrolimus was performed every month. The residual rate tolerated for cyclosporin was 200 to 300 ng / ml during the first two months of pregnancy then the dose was reduced with a target of 100 to 200 ng / ml. The residual rate tolerated for tacrolimus was 10 to 15 mg/l the first two months then reduced to 5 to 10 mg/l. Intrauterine growth retardation was diagnosed in two patients respectively in the 32nd and 36th week of gestation. The fetal heart rate monitoring helped to diagnose acute fetal distress in three patients leading to fetal extraction. The terms of delivery are summarized in Table 2. Delivery was by cesarean section in all cases. Prophylactic antibiotic and anticoagulant was prescribed. The mean duration of pregnancy was 36 weeks gestation and 2 days with extremes of 34 and SA 38 SA + 1 day. In 30% of cases, delivery was premature. Intrauterine growth retardation was present in 20% of cases. There weren't any complications related to the position of the graft during cesarean section. Rhesus negative patient received an injection of anti-D within 72 hours of delivery.

**Table 2:** Outcomes of pregnancy and childbirth

N° Pregnancy	Maternal complication during pregnancy	Term of delivery	Indication for cesarean section	Fetal Alphaimpact
1	-	38 WG, 1 day	AFD	-
2	Toxemia	34 WG	CFD	Hypotrophy
3	Toxemia	35 WG, 3 days	AFD	-
4	Toxemia	37 WG, 6 days	Primary infertility 8 years	-
5	Toxemia, renal dysfonction, infection	36 WG, 4 days	AFD	Hypotrophy
6	Pre-eclampsia	38 WG	Primary infertility 15 years	-
7	-	37 WG	convenience	-
8	-	37 WG	convenience	-
9	-	37 WG, 1 day	Elevated urea	-
10	Pre-eclampsia	38 WG	corporeal scar	-
11	-	37 WG	Primary infertility	-

**WG**: week of gestation, **AFD**: acute fetal distress, **CFD**: chronic fetal distress

The mean birth weight was 2589 with a range of 1600 grams and 3400 grams. In 50% of cases, infants had low birth weight. The neonatal outcome was favorable in all cases. There weren't any malformations reported at birth. Breastfeeding has been proscribed (risk of immunodepression) and contraception by microprogestative was prescribed in all cases. Venous thrombosis in the left superficial radial vein was diagnosed in a patient at 24th day of postpartum requiring hospitalization. The evolution was favorable with anticoagulants at curative doses. The post-partum period was uneventful in all other cases. Immunosuppressive therapy was maintained after childbirth. In three patients, there was switch to azathioprine to mycophenolate mofetil. One patient remained under azathiorpine. During evolution, a patient returned to hemodialysis 6 years and 4 months after delivery and 9 years and 5 months after renal transplantation suites to chronic allograft nephropathy. In all other cases, the evolution of the graft was good with a mean postpartum follow-up of 6 years (extremes of 2 months and 15 years) and a mean post-transplant follow-up of 10 years (extremes of 3 years + 1 month and 16 years + 1 month). Children from these pregnancies are currently aged from 8 months to 15 years. They are in apparent good health. For those of school age, they do not have learning difficulties.

## Discussion

### Nephrological parameters

Chronic glomerular nephropathies (CGN) are recognized in many studies as the most common cause of kidney failure at the origin of the development of chronic hemodialysis in transplanted kidney patients and in whom pregnancy occurred after transplantation. CGN rate was 39.6% in a study by Gill et al. [1], 32% in a study by Areia et al. [2], 52% in a study by Kim et al. [3], 76.1% in a study by Kurata et al. [4], 58.8% in a study by Yildrim et al. [5] and 43.6% in study Rahamimov et al. [6]. In our study, chronic tubulointerstitial nephropathy causing end-stage renal disease was the most frequent (5 cases). The identification of the initial nephropathy is necessary to assess risk of recurrence after transplantation, risk for the graft during pregnancy, or a risk of recurrence in the offspring. It does not seem to be useful for predicting the occurrence of pregnancy in a patient who received a kidney transplant [2,3]. A study conducted in 2009 [1] concluded that patients who had renal failure as a result of diabetes were less likely to conceive than those whose initial nephropathy was CGN. In most studies [5-7], the duration of the dialysis before transplantation has not been evaluated. Long standing hemodialysis correlates with accelerated atherogenesis and is associated with a risk of preterm delivery, growth retardation and fetal distress. This is in agreement with the study of Kurata who considered duration greater than or equal to two years was a risk factor for preterm delivery in a term of less than 35 weeks gestation. But this factor has not been identified as associated with a significant risk [4]. Gill found no significant association between length of dialysis and the probability to conceive. The decrease in the rate of pregnancy among kidney transplant was explained by the wider use of immunosuppressant drugs including cyclosporine in the treatment of CGN prior to renal transplantation [1]. Kidney transplantation is the best treatment for end-stage renal disease. Patients who benefited from a kidney transplant have a longer life, better quality of life and consume fewer health care resources than patients on chronic dialysis [8]. The mean age at transplantation ranged from 23.6 years to 33.4 years, according to studies [1, 3, 6, 7]. The data analysis of the study by Kim and al concluded that younger age at transplantation was a significant predictor for a positive result in the birth of a living child [3].

### Parameters related to pregnancy

Planning, a crucial step, as well as monitoring the pregnancy are the result of close collaboration between the obstetrician and the nephrologist. In general, women at the age of procreation are informed of their possibility of conception and recommended for adequate contraception. If pregnancy is desired, the patient is assessed for renal function with stabilization of associated diseases: high blood pressure equilibration, equilibration of diabetes, treatment of infection. The number of unintended pregnancies remains high (about 50%) in several studies [5,9,10]. Several studies have recommended waiting a period of 2 years after transplantation before allowing patients to conceive [5,6,10-12]. In a recent study (2010), Gorgulu reported that a longer period between renal transplantation and conception is related with a lower rate of premature babies and children born with low birth weight [11]. This attitude was considered too conservative by a study conducted in 2009 by Gill about 530 pregnancies in 483 patients, although the percentage of pregnancies was inversely proportional to time elapsed since transplantation [1]. Kim have recently (2008) concluded that a delay Kidney Transplantation-Conception less than or equal to 1 year was not associated with a higher risk of complications compared with a group of patients whose waiting time before pregnancy was more than one year [3]. Although an optimal interval between transplantation and the design has not yet been established, the National Transplantation Pregnancy Registry (NTPR) and the American Society of Transplantation (AST) suggested waiting 1 year after transplantation, this delay is reasonable to stabilize the renal function and reduce the doses of immunosuppressive drugs [13, 14].

### Occurrence of pregnancies

The incidence of pregnancy after renal transplantation has varied from one study to another. In our series, pregnancies occurred in 12 patients out of 52 female patients having benefited from a kidney transplant and are at the age of procreation (23.07%). This percentage may be underestimated by unnoticed miscarriages. In the study by Kim, only 10% of patients of childbearing age have a pregnancy [3] and the rate was even lower in the study population Gill less than 3% (483 of 1619) [1]. The live birth rates ranged from 55.4% to 90% [1,3,5,9,12].

### Complications during pregnancy

The incidence of hypertension among patients transplanted kidney varies from 60% to 80%. Several factors are involved in the onset of hypertension after renal transplantation: immunosuppressive therapy including corticosteroids and cyclosporine, graft function, the nature of the donor, obesity, alcohol, smoking, presence of a native kidney (increased production of renin) [15]. The diagnosis of preeclampsia can be difficult due to the frequency of hypertension and proteinuria in kidney transplanted patients [16]. Yidirim et al. reported in their study that 15% of patients with worsening hypertension without preeclampsia [5]. Elevation of blood pressure and taking antihypertensive were significantly associated with risk of premature delivery and low birth weight. During pregnancy, the ureter compressed by the gravid uterus expands. The risk of acute pyelonephritis is particularly increased especially in immunodepressed patients. Cruz Lemini [9] reported a frequency of 45.3% urinary tract infections. The gestational diabetes is a complication even more common with the use of steroids but also of cyclosporine and tacrolimus, gestational diabetes was found in a frequency of 5% to 29.3% [5,9].

### Delivery and newborn

**Figure 3:**
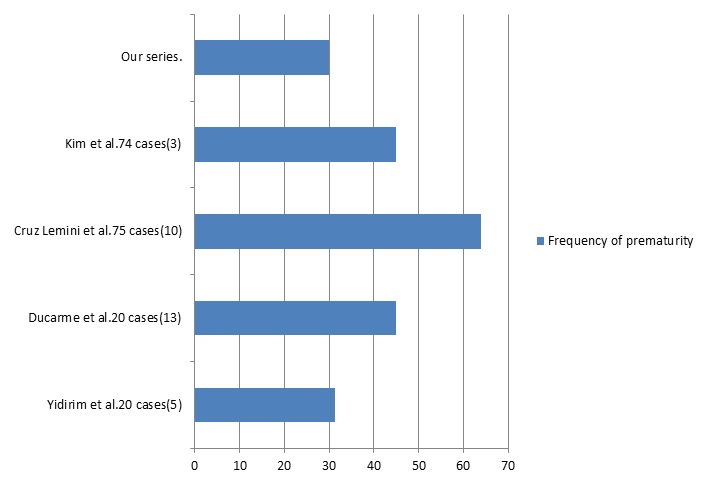
Frequency of prematurity in literature

The lowest mean term at delivery was found in the study of Cruz Lemini 34.2 ± 2.3 weeks of gestation [9]. In most studies, the term at delivery ranged from 35.6 ± 0.3 to 38 weeks of gestation [3, 5, 10]. Premature rupture of membranes (PROM) was noted in 11% to 17%. It has often been responsible for caesarean section. The preterm birth rate was high in this population estimated at 45-60% of cases. In our series, the rate of premature births (30%) is one of the lowest found in the literature (Figure 3). This is related to compliance with international recommendations and the fact that nearly 90% of our patients had a serum creatinine less than 125 μmol / l before conception. In our series, patients who delivered prematurely had hypertension prior to pregnancy. The cesarean rate ranges from 40% to 100%, is probably not justified solely by obstetric indications [2,9,12]. Low birth weight was reported in 40% to 50% [12]. Intrauterine growth retardation is associated with the use of cyclosporine and prematurity.

### Long-term follow-up

Most studies have shown that graft survival in patients who had a pregnancy was equivalent to that of patients who were not pregnant [6,17]. The NTPR found that graft loss in patients who had a pregnancy fluctuated between 4% and 14% within two years after delivery. These rates are similar to those observed in renal transplant who were not pregnant [18]. Data on children from these special pregnancies are reassuring. Nulman compared 39 children exposed in utero to cyclosporine in kidney transplanted patients with a control group of 38 children. No significant differences were found in neurocognitive and behavioral development [19].

## Conclusion

Renal transplantation allows patients suffering from end-stage renal disease improving quality of life and represents a legitimate hope for all those who want to conceive. Pregnancy in kidney transplanted patients is a high risk pregnancy because of the higher incidence of low birth weight, prematurity and intrauterine growth retardation. A favorable outcome of pregnancy and the preservation of renal graft is possible through: planning pregnancy (stable renal function and hypertension balanced), monitoring conducted in coordination with the gynecologist and the nephrologist and childbirth in a maternity 3^rd^ level. For a better understanding of the particularities of pregnancy in kidney transplanted patients in Tunisia, the establishment of a national registry collecting information from all centers of kidney transplant, all services of Gynecology and Obstetrics and free practice physicians is necessary to support findings by statistics on a more significant number of patients.

### What is known about this topic

- Motherhood has long been no
- t recommended for kidney transplanted women by fear of deleterious effects on graft.

### What this study adds

- Under certain conditions, pregnancy in kidney graft is possible;
- Our study showed that a favorable outcome of pregnancy and the preservation of renal graft are possible through: planning pregnancy, monitoring conducted in coordination with the gynecologist and the nephrologist and childbirth in a maternity of 3^rd^ level.

## Competing interests

The authors declare no competing interests.
